# The Analysis of Deformability, Structure and Properties of AZ61 Cast Magnesium Alloy in a New Hammer Forging Process for Aircraft Mounts

**DOI:** 10.3390/ma14102593

**Published:** 2021-05-16

**Authors:** Anna Dziubińska, Piotr Surdacki, Krzysztof Majerski

**Affiliations:** Faculty of Mechanical Engineering, Lublin University of Technology, 20-618 Lublin, Poland; piotr.surdacki@pollub.pl (P.S.); k.majerski@pollub.pl (K.M.)

**Keywords:** magnesium alloys, deformation, hammer forging, aircraft mounts, FEM, industrial research, structure, mechanical properties

## Abstract

This article presents the analysis of the deformability, structure and properties of the AZ61 cast magnesium alloy on the example of a new forging process of aircraft mount forgings. It was assumed that their production process would be based on drop forging on a die hammer. Two geometries of preforms, differing in forging degree, were used as the billet for the forging process. It was assumed that using a cast, unformed preform positively affects the deformability of hard-deformable magnesium alloys and flow kinematics during their forging and reduces the number of operations necessary to obtain the correct product. Numerical analysis of the proposed new technology was carried out using DEFORM 3D v.11, a commercial program dedicated to analyzing metal forming processes. The simulations were performed in the conditions of spatial strain, considering the full thermomechanical analysis. The obtained results of numerical tests confirmed the possibility of forming the forgings of aviation mounts from the AZ61 cast magnesium alloy with the proposed technology. They also allowed us to obtain information about the kinematics of the material flow during forming and process parameters, such as strain intensity distribution, temperatures, Cockcroft–Latham criterion and forming energy. The proposed forging process on a die hammer was verified in industrial conditions. The manufactured forgings of aircraft mounts made of AZ61 magnesium alloy were subjected to qualitative tests in terms of their structure, conductivity and mechanical properties.

## 1. Introduction

Lately, an increased interest in magnesium alloys has been noticeable [1,2,3,4,5,6], especially in aviation and the automotive industry [7,8,9]. Decreasing the structure mass has become a major priority in many branches of the industry. This is why magnesium-based elements made of light metal alloys are increasingly explored in machine construction [10].

However, magnesium is a reactive metal and very susceptible to corrosion, especially in environments containing chloride ions, limiting the application area of magnesium alloys. For this reason, it is necessary to protect the surface of magnesium components by applying additional paint coatings, conversion coatings or electrochemical coatings, or by using anodizing processes and vapor deposition of coatings [11,12,13,14].

In aviation, a wide range of construction elements is used, e.g., gearbox, engine, wing, hull plating, door, wheels, landing gear, cockpit panels and seat elements [15]. For example, in Boing 727 c.a. 1200 elements are made of magnesium. As far as the automotive is concerned, magnesium alloys are used in, among others, producing engines, bodywork, cylinder head covers, seat and sunroof frames and pedal support brackets and stems [7,16]. In particular, heat treatment of magnesium alloys is used in aerospace and automotive applications. This is important because high mechanical properties can be achieved for critical aerospace and automotive parts. Annealing, supersaturation and aging of magnesium alloys are used [2,17,18,19].

Magnesium alloys are also found in household items. Using magnesium, the density, of which is 1.74 g/cm^3^, allows to significantly decrease the product’s weight, even by 30%. The factors limiting the usage of magnesium alloys include low corrosion resistance, flammability, lower strength, specific forming conditions due to a narrow range of temperature parameters and sensitivity to strain, which results in high-cost of metal forming and mechanical machining [8,20,21].

The magnesium alloys are the most widely used as-cast alloys [22,23,24,25]. This is conditioned by their availability on the market, well-developed casting technology and lower price. Unfortunately, the quality of the obtained castings in terms of their strength and functional properties is insufficient due to the occurrence of the following casting defects: heterogeneity of the structure, coarse grain structure, blisters, porosities, contraction cavities, femoral stems and other defects, which decrease the durability of the obtained castings.

The necessity of using castings is forced by the low availability of magnesium forgings, which would significantly increase the mechanical and functional properties of the final product and decrease the production cost. Despite the beneficial mechanical properties, using magnesium alloys for forming accounts for only 1% of the annual production of magnesium in the world, which is related to the limited plasticity of these magnesium alloys.

Metal forming of magnesium alloys proves even more difficult due to the narrow range of temperature parameters and sensitivity to the strain. Therefore, forming magnesium alloys is carried out in high-temperature on forging machines with low operating speeds while maintaining isothermal temperature conditions during deformation. Currently, the few companies that have implemented the technology of forging magnesium alloys on specialized hydraulic presses with tool heating systems include Otto-Fuchs and Weisensee Warmpressteile from Germany and KUMZ from Russia.

Some casting alloys are also formed, including by forging [26,27]. This way, products from castings have better mechanical and functional properties, which results in the uniformity and fragmentation of the casting structure. Standard shapes of billets available on the market are used for forging magnesium alloys, and the process is carried out in isothermal conditions on specialized presses equipped with heating systems. Low production capacities are caused mainly by the recommended forging technology on very slow presses, and using complicated heating installations mounted in the tooling contributes to high production costs and low attractiveness of products compared to, e.g., aluminum.

The die forging of magnesium alloys on forging equipment, such as forging hammers without specialized heating systems, would reduce production costs and increase productivity. Unfortunately, using standard shapes of castings for forging magnesium alloys, particularly hard-deforming, on traditional hammers does not guarantee correct forgings without cracks. Often the first cracks inside the material appear during the initial forging when forging is formed. Further processing of the forgings in the finishing impression reveals a defect in the form of cracks, which disqualifies the product. The solution to this problem could be using the ready-made preforms, which are cast to mirror the forgings as closely as possible. Theoretically, in terms of costs, using preforms obtained by the casting should be more advantageous than using billets, and consequently, should be an interesting alternative. In this case, the production process of the charge is shortened by the operations related to the extrusion of the billets themselves and the initial forging of the preforms. It seems that such a solution may also have several valuable advantages. The mechanical properties of the forgings obtained from the billets are comparable for both technologies, and in many cases, favor those cast due to their low anisotropy. There are, however, differences in the structure of the forgings made of ingot compared to using wrought billet forging. The forging made of an extruded billet receives in some sections a fibrous structure, which results in different values of strength properties depending on the direction of the fibers in the forging. In some cases, this may be disadvantageous from the point of view of the distribution of the mechanical properties in the product. A more fragmented and homogeneous structure in the entire volume of the product favor forgings made of cast materials.

It was assumed that using a preform cast as close as possible to the shape of the forging in the plane of the die division would have a positive effect on the kinematics of metal flow, especially when deforming less deformable grades of magnesium alloys, which would allow obtaining a correct product without defects with a more precise shape and dimensions. By limiting the number of operations needed to produce the forging, greater material and energy savings can be achieved.

Therefore, it was considered advisable to test this concept and develop new technology for forging magnesium alloys from cast preforms. The AZ61 magnesium alloy with good strength properties, which is of interest to the aerospace and automotive industries, was selected for the study. The article analyses the deformability, structure and properties of the AZ61 cast magnesium alloy in the form of preforms used in the new hammer forging process of the forgings of the aircraft mounts.

## 2. Research Methodology

### 2.1. Assumptions of the New Technology and Numerical Simulations

**Figure 1:**
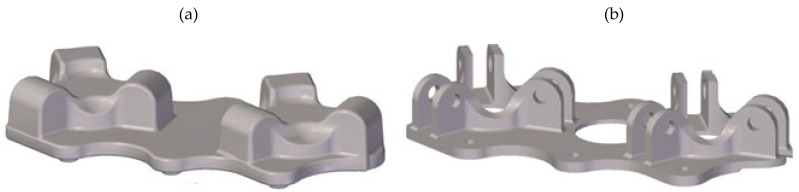
3D models of the forging of aircraft mount from AZ61 alloy (**a**) and of the finished aircraft mount (**b**).

**Figure 2:**
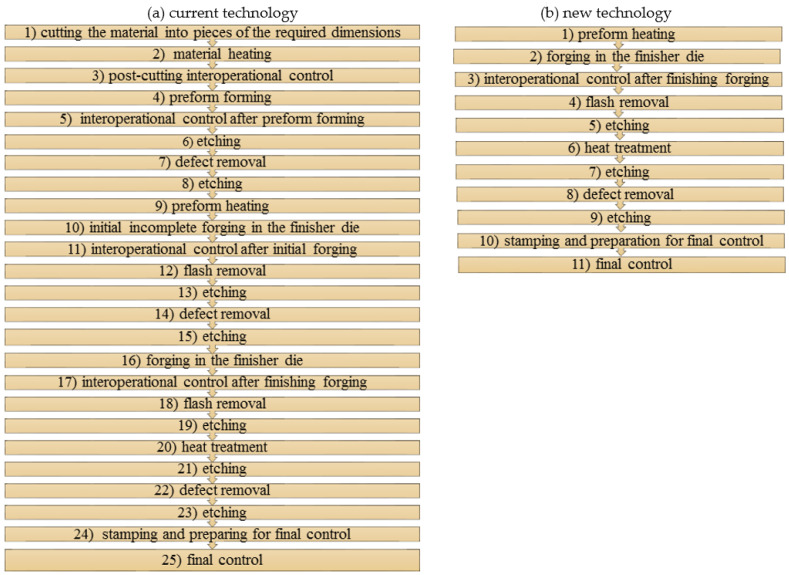
Scheme of the currently applied, multi-stage forging process for high-strength magnesium alloy from an extruded billet (**a**) and the new forming process (**b**).

The research concentrated on a new forming process of forging an aircraft mount (Figure 1a) manufactured from a billet in the form of a sand-cast preform from a high-strength AZ61 grade magnesium alloy. Currently, aircraft mounts presented in Figure 1b are manufactured by machining from cast elements or by multi-stage forging from wrought billet [28,29,30,31]. This technology is particularly time, labor, and energy-consuming. Moreover, it generates significant material waste, while the quality of the finished product is low. Among other manufacturing methods, there is a die forging of a wrought billet. In this method, limits to the usage occur since it is difficult to produce elements from less plastic magnesium alloys. The process is conducted in many stages (Figure 2a), leaving a significant stock allowance. Approximately 50% of the forging mass is technological waste created in several forging operations and numerous heating operations. In the die forging of mounts from less plastic magnesium alloys, it is often necessary to obtain additional dies for initial forging. This technology is also very material-, labor- and energy-consuming, as well as limitedly effective.

It was assumed that in the new process of forging an aircraft mount from AZ61 grade alloy, the geometry of the preform would be similar to the forging, especially in terms of the outline in the area of die division. Using a not-wrought preform positively influences the flow kinematics and deformability of the material during forging. Using a billet with precise/accurate dimensions in the form of cast preform allows reducing material waste compared to the process currently used in the industry, that is, forging from an extruded billet. The process is realized in one forging operation in a finishing die, using typical forging machines, die hammers and inexpensive tool heating methods (furnace, gas burner). It is also worth mentioning that using a ready-made cast forging for the forging process limits the number and duration of operations required for the forging to be obtained, which influences the effectiveness and decreases the labor consumption of the process. Elements subjected to metal forming following the new technology are of better quality, resulting from a better macro- and microstructure, whereas its surfaces are smoother, which, in turn, enhance the functional and mechanical properties of the product compared to the ones made from cast only.

**Figure 3:**
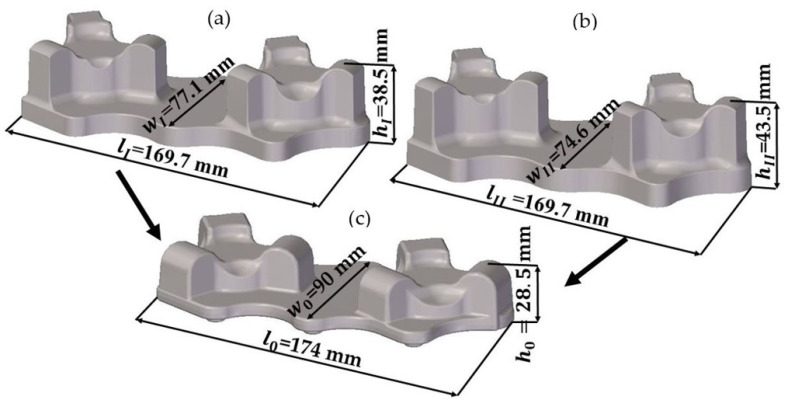
Model 3D of the parts: (**a**) variant I-preform with a smaller forging ratio, (**b**) variant II-preform with a higher forging ratio, (**c**) forging.

**Figure 4:**
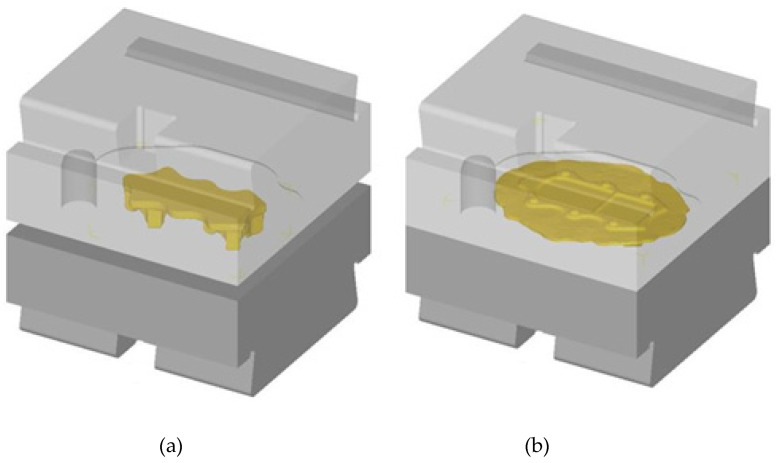
An exemplary process of forging an aircraft mount from AZ61 magnesium alloy, conducted in a die hammer for the preform with a smaller forging ratio: (**a**) beginning of the process, (**b**) end of the process.

The numerical analysis of forging the aircraft mount in a hammer from AZ61 grade alloy was performed using the finite elements method (FEM) in Deform 3D ver. 11.0 software (Scientific Forming Technologies Corporation, Columbus, Ohio, United States). FEM simulations were conducted with the spatial strain state and applying the full thermomechanical model. For the new process, two preforms geometries were designed, with a more and less significant forging degree, being the ratio of the height of the billet h_0_ to the height of the deformed forging. The forgings differ mainly in height and thus in the degree of forging and the cross-sectional dimensions in the division plane. The degree of forging, i.e., the ratio of the height of the forging preform to the height of the forging product for the first variant was h_I_/h_0_ = 38.5/28.5 = 1.35, and for the second variant, h_II_/h_0_ = 43.5/28.5 = 1.52. The scheme of the geometry of the preforms and the forging is shown in Figure 3. It was assumed that the new forming process conducted from the cast preform with a smaller and higher forging ratio would be realized in one forging operation in the finishing impression. Figure 4 presents an exemplary forging process. It was assumed in the simulations that the preform-shaped billet is heated to 400 °C [32], whereas the die temperature during the process is constant and equal 250 °C [32]. The material was divided into 150,000 tetragonal elements. The material model of the AZ61 magnesium alloy cast in the sand casts was developed based on its own plastometric tests on a DIL 805A/D strain dilatometer. The plastic strain was introduced to the program in table form and depends on the temperature in the range 240–440 °C, strain rate from 0.01 s^−1^ to 10 s^−1^ and strain values in the range 0–1.

The forging process for manufacturing magnesium alloy aircraft bracket is performed with a hammer described by the impact energy of 36 kJ and the weight of the dropping part of 1200 kg. The heat transfer coefficient between the workpiece and the environment was assumed to be 0.03 kW/m^2^K and between workpiece and tools 4.5 kW/m^2^K [33]. The friction conditions between the formed material and the tools were described with the constant friction model. The friction factor describing the contact between the AZ61 magnesium alloy and steel with graphite lubrication was set to m = 0.24 [34].

### 2.2. The Material Used for the Tests and the Conditions for the Experimental Tests for Forging of Aircraft Mounts Forgings

**Figure 5:**
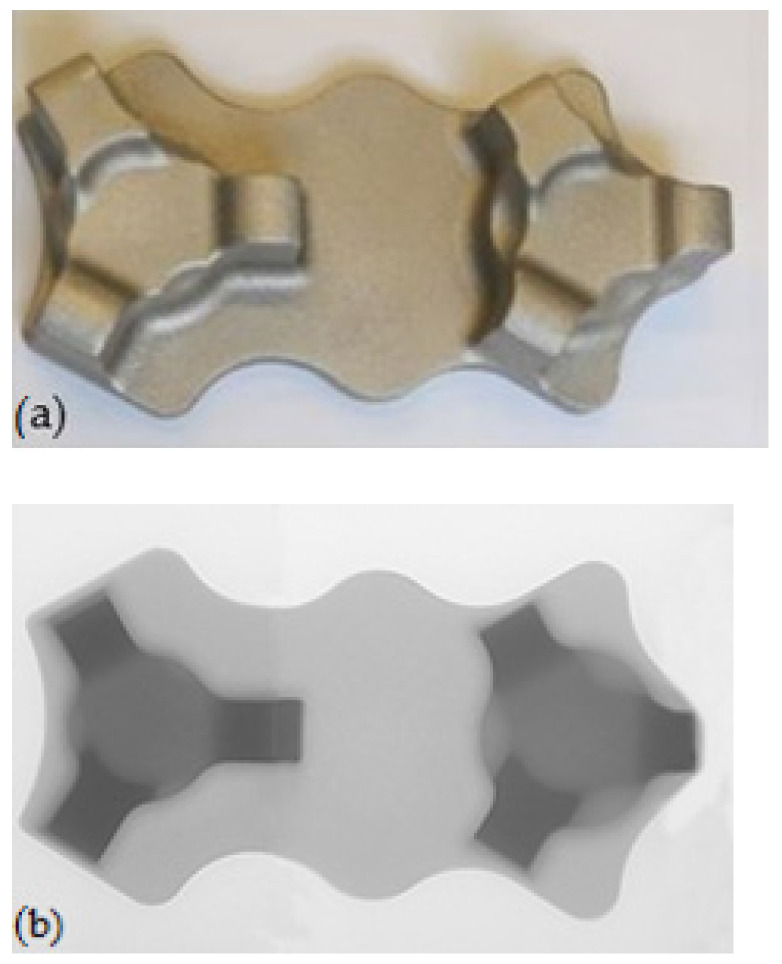
Cast preforms: (**a**) lower degree of forging, (**b**) X-ray of a preform.

AZ61 magnesium alloy cast into sand molds was used for the experimental tests. The chemical composition of the AZ61 alloy is shown in Table 1. Preforms form-shaped casts (Figure 5) were made by NEOCAST Lightweight Metal Technologies (Cracow, Poland). All castings were subjected to the annealing by heating a furnace to the temperature T = 415 °C in a protective argon atmosphere, heating at T = 415 °C for 24 h, and then cooling in air. The annealing temperature was selected based on testing the hardness and microstructure of castings annealed at different temperatures.

**Figure 6:**
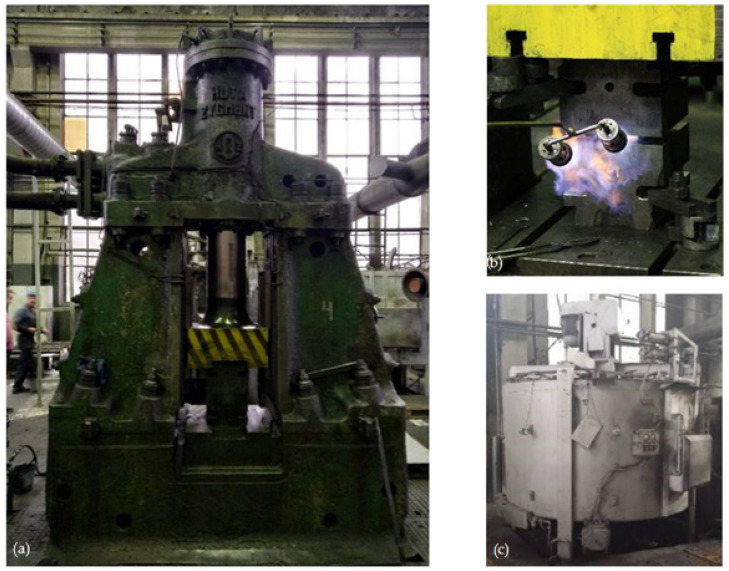
Machines used during tests in industrial conditions: (**a**) MPM 3150 drop hammer, (**b**) gas burner, (**c**) electric furnace.

**Figure 7:**
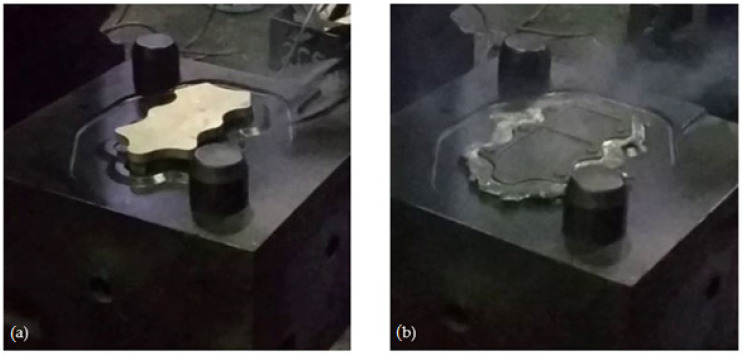
Forging in a finishing impression for a cast preform with a lower degree of forging: (**a**) start of the process, (**b**) end of the process.

The experimental tests of the new process of forming forgings of aircraft mounts from AZ61 alloy castings were carried out in industrial conditions at ZOP Co. Ltd. FORGING PLANT (Świdnik, Poland). All forging tests were performed on an MPM 3150 die hammer (Huta Zygmunt, Poland) (Figure 6a) using a set of dies shown in Figure 7 and the conditions assumed in computer simulations (Section 2.1). The dies were heated in the furnace to the temperature of 250 °C, and during the tests, their temperature was maintained using gas burners (Figure 6b). The preforms were heated to the forging temperature of 400 °C in a PEO-A1-type rotary electric furnace with forced air circulation (ELTERMA Świebodzin, Poland) (Figure 6c). After obtaining the appropriate thermal conditions, the preforms were forged in a single operation in a finishing impression (Figure 7). Correct forgings of aviation mounts were trimmed from the flash using a saw and then etched in five baths with the parameters presented in Table 2. Then, the forgings were heat-treated in the form of supersaturation at T = 415 °C for 6 h and cooling in water and aging in temperature T = 175 °C for 24 h and cooling in air.

### 2.3. Types of Qualitative Tests Performed for the Formed Forgings

**Figure 8:**
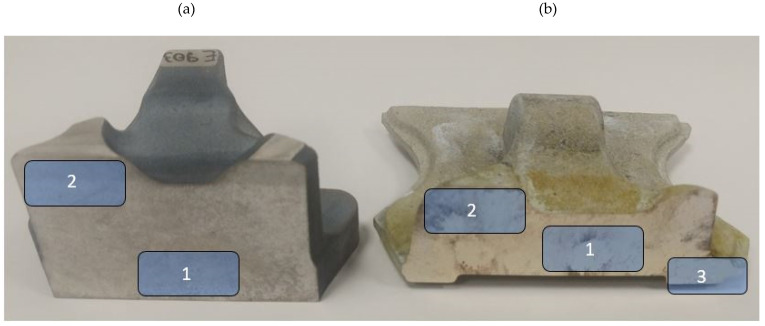
Microstructure test areas, (**a**) preform, (**b**) forging.

To assess the quality of the formed AZ61 alloy forgings of aircraft mounts obtained during industrial tests, the structure, electrical conductivity and mechanical properties were tested. The macrostructure tests were carried out in the cross-section of the mounting rib (Figure 8) for cast preforms, homogenized cast preforms, forgings after forging as well as forgings after forging and heat treatment, respectively. First, cross-sections were made, which were subjected to the process of grinding and polishing. Initially, grinding was applied on a SiC-coated abrasive disc with a grain size of 400 for 3 min. Then, polishing with a diamond suspension of 9 µm was applied for 3 min. The next step was polishing using a diamond suspension with a grain size of up to 3 µm for 3 min. Then polishing was carried out with colloidal silica with a grain size of 0.05 µm for 3 min. Between each step, the samples were rinsed substantially with alcohol to counteract surface oxidation. After the polished sections were made, the samples were etched by immersion and gentle stirring for 5–15 s in a solution of 100 mL ethanol, 10 mL distilled water, 10 mL acetic acid, and 5 g picric acid etchant.

Microstructural examinations were carried out using the NIKON MA200 optical microscope (Tokyo, Japan). Quantitative microstructure analysis was performed using Image-Pro 10 software. The analysis was carried out both in the central and near-surface areas of the samples, as shown in Figure 8. The chemical composition analysis in micro-areas was carried out by the EDS method using a Phenom ProX scanning electron microscope equipped with a CeB_6_ crystal source and an SDD-type EDS detector.

The next performed qualitative tests were tests of specific electrical conductivity. They were carried out to evaluate the structure of the material after forging and after heat treatment. Conductivity measurements were made on the metallographic polished sections analyzed during the microstructure tests. The conductivity of the samples was measured with a SIGMATEST model 2.069 (Ferster Instruments Incorporated, Pittsburgh, PA, USA). The measurements were made at an ambient temperature of 21 °C.

Strength tests were carried out on samples made of heat-treated cast preforms and on forgings of mounts. The geometry of the samples and the test procedure were following ISO 6892-1. A Shimadzu AG-X plus 20KN testing machine (Kyoto, Japan) was used for the tests, equipped with a longitudinal extensometer to measure deformations and to control the test speed in the feedback loop. The tests were carried out under controlled conditions at 21 °C. The speed of the test was variable. In the elastic range, the strain was controlled using the signal from the extensometer, and the speed was 0.025%/s. In the range of plastic flow, the traverse displacement speed was 0.9 mm/min.

Hardness measurements were made using the Vickers method with the Future-tech FM800 hardness tester (Future-Tech Corporation, Kawasaki, Japan). Measurements were made on the HV0.5 scale following PN-EN ISO 6507-1: 2006

## 3. Results and Discussion

### 3.1. Results of Numerical Simulations

**Figure 9:**
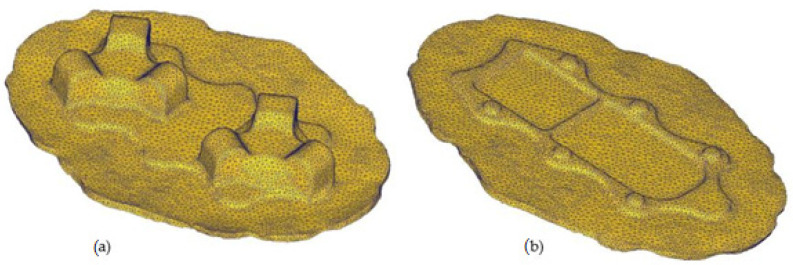
FEM-simulated shape of aircraft bracket forging: (**a**) top, (**b**) bottom.

The theoretical analysis results confirmed the possibility of forming the forging of the aircraft mount from AZ61 magnesium alloy from cast preforms of an assumed geometry. Figure 9 shows the correct shape of the forging from the FEM simulation. The formed elements are correct in shape, which confirms the proper design of the process and good deformability of the cast AZ61 magnesium alloy under the assumed temperature conditions and the assumed geometry of the preforms.

**Figure 10:**
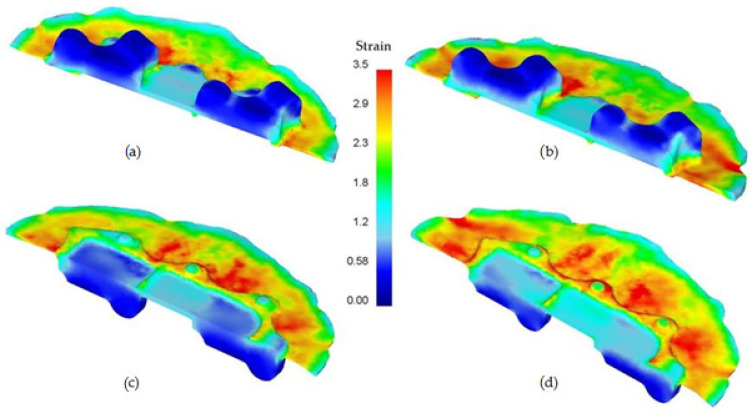
Distribution of the effective strain in the cross-section of the AZ61 aircraft mount: (**a**) lower degree of forging–top of the forging, (**b**) a higher degree of forging–top of the forging, (**c**) lower degree of forging–bottom of the forging, (**d**) higher degree of forging–bottom of the forging.

The numerical analysis results also provided information on the important parameters of the process, such as strain intensity, distribution of temperature and Cockcroft–Latham damage criterion and the forming energy. The distribution of the effective strain in the formed forging of the aircraft mount is shown in Figure 10. It can be observed that in both of the formed forgings (with smaller and higher forging degrees), the distribution of effective strain is similar and heterogeneous in the entire product. The highest values of this parameter occur in the surface areas of the bottom of the forging and in the flash, which is typical for die forging processes and for hammer forging.

**Figure 11:**
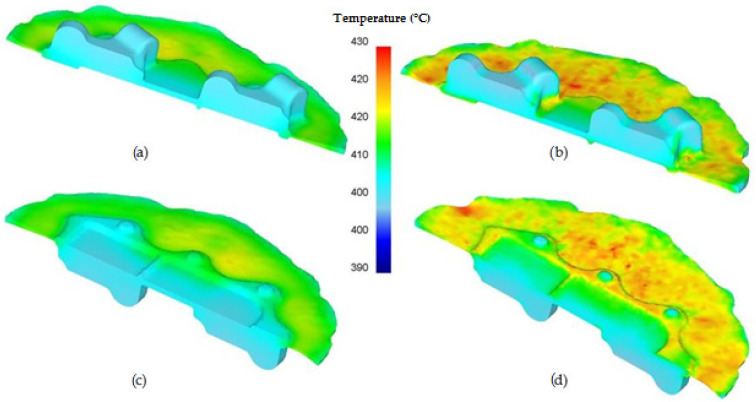
Distribution of temperature in the cross-section of the AZ61 aircraft mount: (**a**) lower degree of forging–top of the forging, (**b**) higher degree of forging–top of the forging, (**c**) lower degree of forging–bottom of the forging, (**d**) higher degree of forging–bottom of the forging.

Additionally, the distribution of temperature in the formed forgings (Figure 11) was analyzed. It can be observed that in the areas of high strain, temperatures exceeding 410 °C occur for a preform of a lower degree of forging and around 420 °C for the other version of the preform. It is a typical distribution of those parameters in the die forging processes and does not negatively influence the quality of the products as the flash is removed. In other areas, temperatures do not exceed 400 °C.

Along with the analysis of the parameters discussed above, the risk of cracking in the forging was researched. For the theoretical analysis of this phenomenon, the Cockroft–Latham (C–L) failure criterion [35] in a modified form implemented in the Deform 3D program. This program determines the places at risk of cracking based on this criterion expressed by the formula:

(1) $$\int_{0}^{\varepsilon_{p}}\frac{\sigma_{\max}}{\sigma_{H}}d\varepsilon =C_{1}$$

where σ_max_-maximum principal stress, σ_H_-equivalent stress according to Huber’s hypothesis, 𝜀-strain intensity, C_1_-integral value.

**Figure 12:**
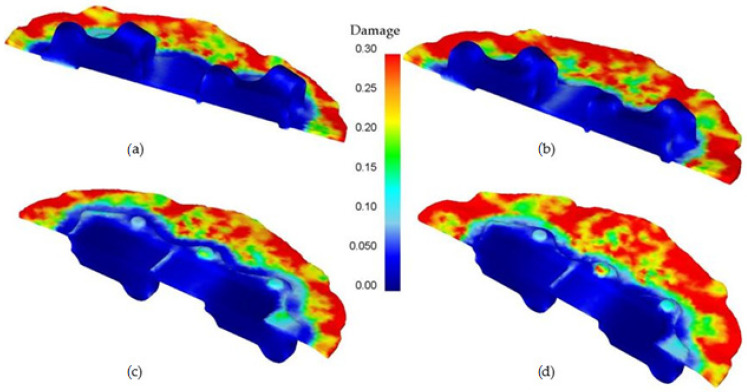
Distribution of the Cockcroft–Latham damage criterion in the axial section of the AZ61 aircraft forging: (**a**) lower degree of forging–top of the forging, (**b**) higher degree of forging–top of the forging, (**c**) lower degree of forging–bottom of the forging, (**d**) higher degree of forging–bottom of the forging.

The C-L criterion assumes that when the work done by tensile stresses in uniform tension reaches a certain critical value C_1_ = C_CL_, plastic fracture of the material occurs. The results are shown in Figure 12. The distribution of the Cockcroft–Latham damage criterion indicates that the highest risk of cracking is on the circumference of the flash. This phenomenon remains following the industrial practice for hard-deformable materials, that is, among others, the AZ61 alloy. For this reason, radial cracks often occur on the circumference of the flash.

**Figure 13:**
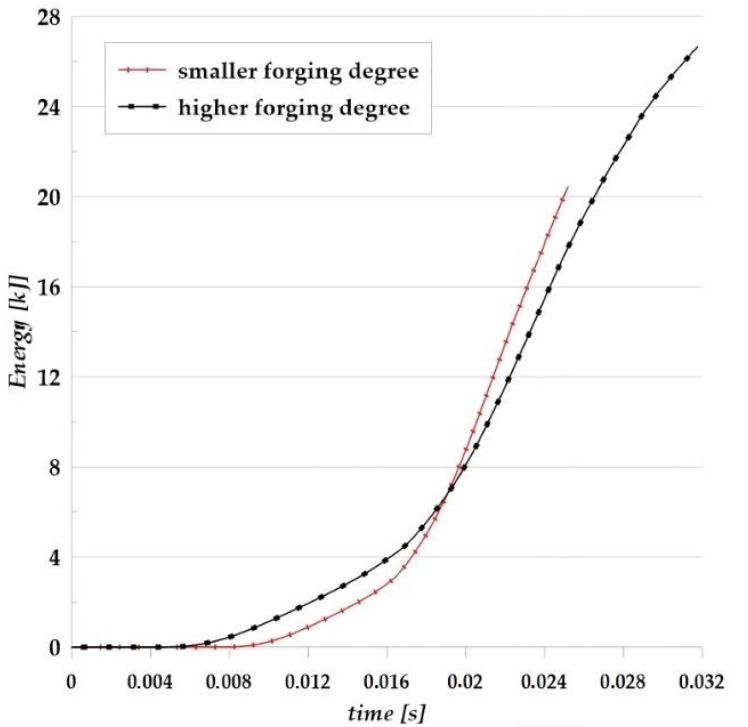
Scheme of the forging energy of the AZ61 aircraft mount forging for the two analyzed preforms.

FEM analysis allowed to determine the forming energy (Figure 13) occurring during forging in the die hammer in a finishing die from two designed preforms. The maximum energy needed to obtain the forging from the first (smaller) variant was approximately 21 kJ, and for the second variant, approximately 27 kJ. This justifies the conclusion that the hammer on which the process was carried out was appropriately selected.

Table 3 shows the comparative analysis performed in volume and material losses of forging from cast preforms with the technology of forging from an extruded rod. Forming the mount forgings from cast preforms was characterized by lower material consumption by about 47% for variant I and about 18% for variant II compared to the currently used forging technology directly from the rod.

### 3.2. The Results of Experimental Tests

**Figure 14:**
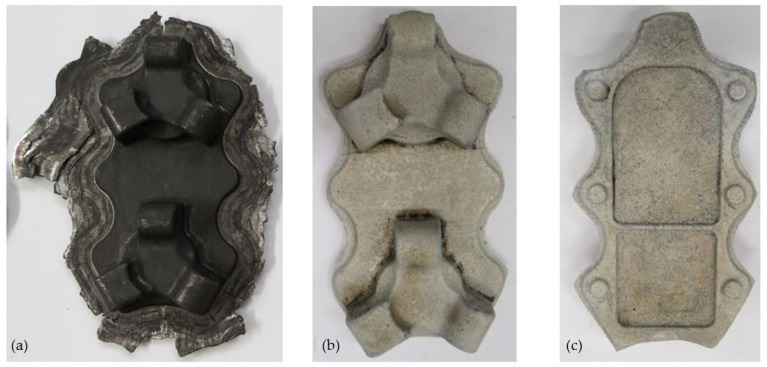
Forging obtained for a preform with a lower degree of forging (variant I): (**a**) after forging, (**b**) top view after trimming, (**c**) bottom view after trimming.

**Figure 15:**
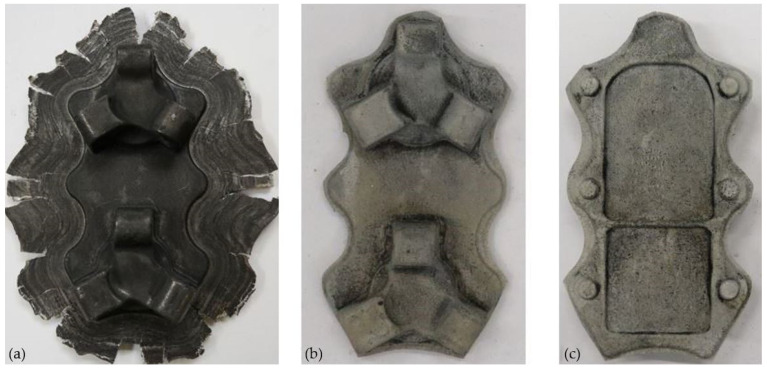
Forging obtained for a preform with a higher degree of forging (variant II): (**a**) after forging, (**b**) top view after trimming, (**c**) bottom view after trimming.

The results of experimental tests carried out in industrial conditions confirm the good deformability of the AZ61cast magnesium alloy and the possibility of producing aircraft mounts from both geometry variants of the cast-shaped preforms. Figure 14 and Figure 15 show the forgings obtained for both variants of the preforms: a lower degree of forging (Figure 14) and a higher degree of forging (Figure 15). After the visual inspection of the forgings obtained after trimming the flash and etching (Figure 14b,c and Figure 15b,c), no laps or other surface defects were found, proving that the correct products were obtained.

### 3.3. The Results of Qualitative Research

**Figure 16:**
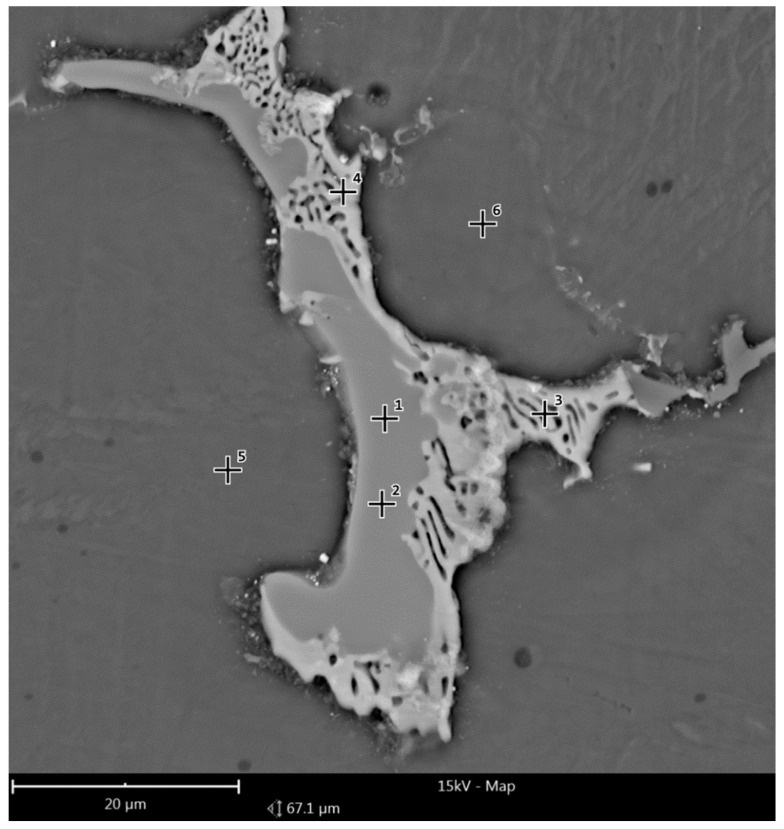
Reference second area of analysis marked on Figure 8 of the cast preform variant I cross-section used to analyze the chemical composition in micro-areas.

**Figure 17:**
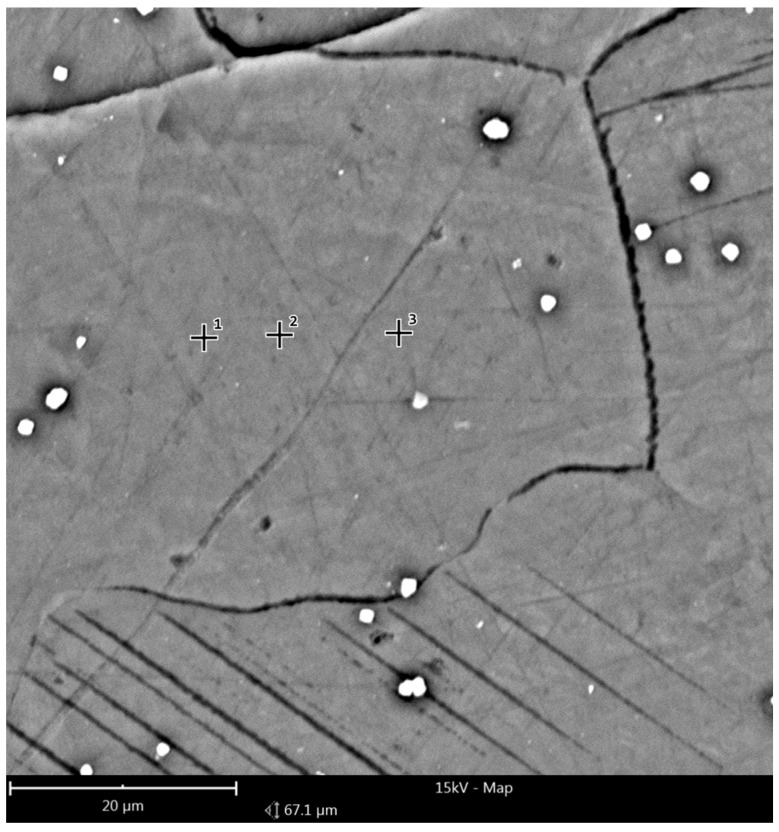
Reference second area of analysis marked on Figure 8 of the cast preform variant I cross-section after homogenization used to analyze the chemical composition in micro-areas.

Figure 16 and Figure 17 show the reference area of the preform variant I cross-section; (a) as-cast, (b) homogenized, used to analyze the chemical composition in micro-areas. The figure shows the points where the microanalysis was performed. The measurement results are summarized in Table 4.

The microanalysis results indicated a significant heterogeneity of the chemical composition of the preform in the as-cast state, which was characteristic of this type of alloy. Three basic regions were distinguished, regions of different composition: the zone of Mg solid solution with low content of alloying additives, precipitations of the intermetallic phase (most likely Mg17Al12) and eutectic regions. As a result of the homogenization process, the chemical composition became homogeneous and was close to the nominal one over the entire tested cross-section.

**Figure 18:**
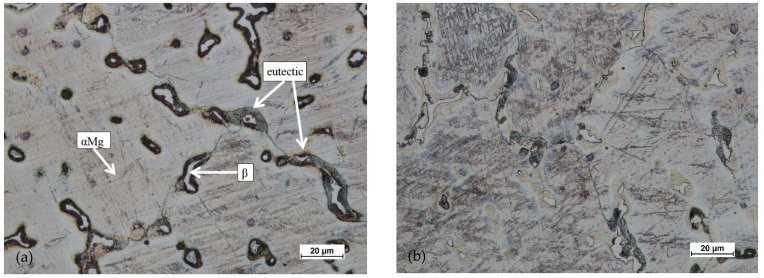
Microstructure for the cast preform, variant I: (**a**) first area of analysis marked on Figure 8, (**b**) second area of analysis marked on Figure 8.

**Figure 19:**
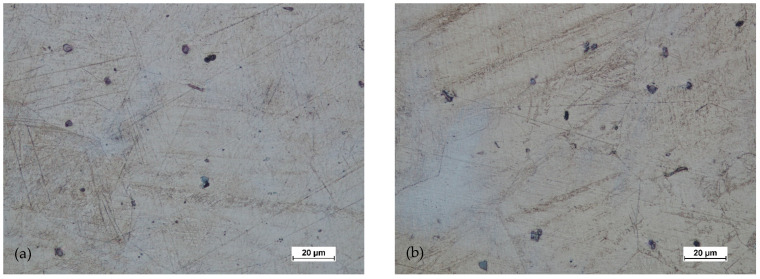
Microstructure for the cast preform, variant I after the homogenization process: (**a**) first area of analysis marked on Figure 8, (**b**) second area of analysis marked on Figure 8.

**Figure 20:**
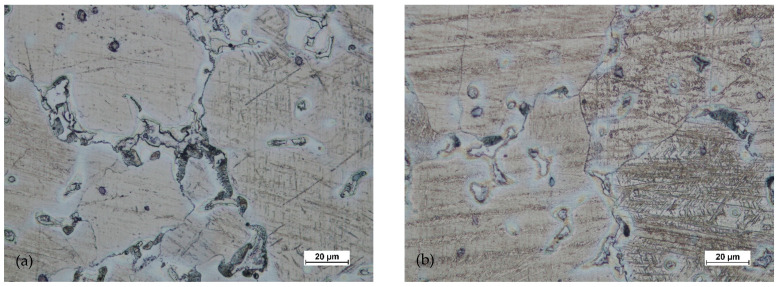
Microstructure for the cast preform, variant II: (**a**) first area of analysis marked on Figure 8, (**b**) second area of analysis marked on Figure 8.

**Figure 21:**
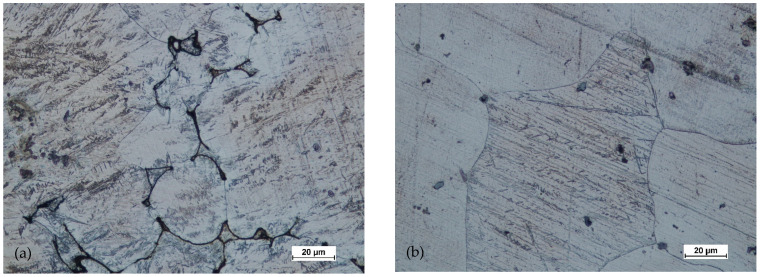
Microstructure for the cast preform, variant II after the homogenization process: (**a**) first area of analysis marked on Figure 8, (**b**) second area of analysis marked on Figure 8.

Based on studying the microstructure of the cast preforms in their raw state, the structures shown in Figure 18, Figure 19, Figure 20 and Figure 21 were obtained.

All raw castings had a homogeneous structure throughout the tested cross-section. This structure was characteristic of this as-cast alloy and was dendritic. It consisted of chain precipitates of intermetallic phased, most of which were β phase (Mg17Al12) surrounded by eutectic precipitated. The alloy matrix was a solid solution of αMg. After homogenization of the castings, grain growth was visible, with the dissolution of most of the precipitates in the alloy matrix.

**Figure 22:**
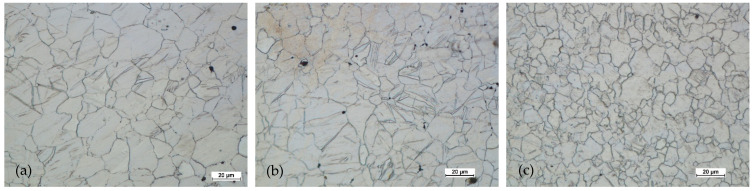
Microstructure for the forging obtained from the preform I: (**a**) first area of analysis marked on Figure 8, (**b**) second area of analysis marked on Figure 8, (**c**) third area of analysis marked on Figure 8.

**Figure 23:**
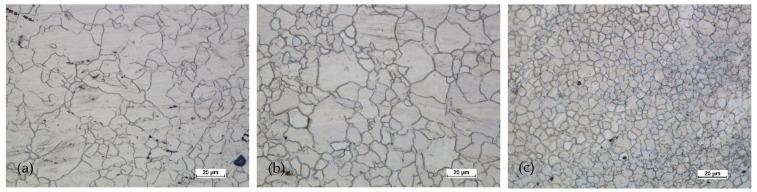
Microstructure for the forging obtained from the preform II: (**a**) first area of analysis marked on Figure 8, (**b**) second area of analysis marked on Figure 8, (**c**) third area of analysis marked on Figure 8.

**Figure 24:**
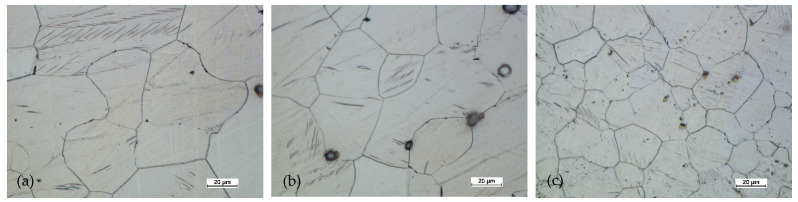
Microstructure for the forging obtained from preform I after forging and heat treatment: (**a**) first area of analysis marked on Figure 8, (**b**) second area of analysis marked on Figure 8, (**c**) third area of analysis marked on Figure 8.

**Figure 25:**
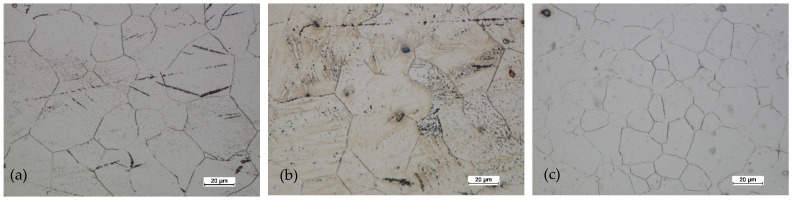
Microstructure for the forging obtained from preform II after forging and heat treatment: (**a**) first area of analysis marked on Figure 8, (**b**) second area of analysis marked on Figure 8, (**c**) third area of analysis marked on Figure 8.

Figure 22, Figure 23, Figure 24 and Figure 25 show the microstructures for forgings of aircraft mounts obtained in the forging process with a die hammer.

After the preform of the first variant was hammered, the microstructure had a significantly finer grain both in the central zone and near the surface. In the third area, with the highest intensity of deformation, the grain was the finest. The grain size was relatively uniform (for AZ61 magnesium alloy). The twin deformations were visible.

In the case of forging a higher preform (second variant), the situation was analogous. The greatest grain refinement occurs in the third area, and in areas 1 and 2, the grain sizes were less homogeneous compared to the lower preform. However, the difference was not significant. The grain size differences were acceptable. The shape of the grains in all studied areas indicated that the processes of dynamic recovery and recrystallization were significantly advanced. When comparing the structural homogeneity obtained in the considered variants of the preforms, it should be noted that, although the most favorable microstructure was obtained for area 3 of the preform II, the differences in individual areas were smaller in the case of the I variant preform. This may indicate that a high degree of forging promotes fragmentation and homogeneity. However, the differences in the individual areas indicate that the distribution of deformations in the II variant forging was less homogeneous.

Figure 24 and Figure 25 show the microstructures of the forgings after forging and heat treatment.

After heat treatment, the grain regrowth was noticed. However, it was not as significant as after homogenization. Lenticular secondary precipitated were visible (discontinuous). In all areas, the grain size was similar, and its shape was regular.

A quantitative assessment of the microstructure was carried out in the areas analyzed on the surface of preforms and forgings. Table 5 summarizes the average values of the grain size in individual areas marked in Figure 8.

The as-cast grain size was significant, mainly due to undeveloped grain boundaries in the dendritic structure. The grain was still relatively large after homogenization. As a result of forging, the grain broke down, and due to recrystallization, the average grain size dropped significantly, especially in areas with high deformation value (3 area), as indicated by the results of numerical simulations. For the variant with a higher degree of deformation, the average grain size was smaller in all areas.

The use of heat treatment increased the grain size in all areas, particularly for variant I. The obtained results indicate that using high deformation values had a positive effect on the microstructure obtained at the end of the process.

**Figure 26:**
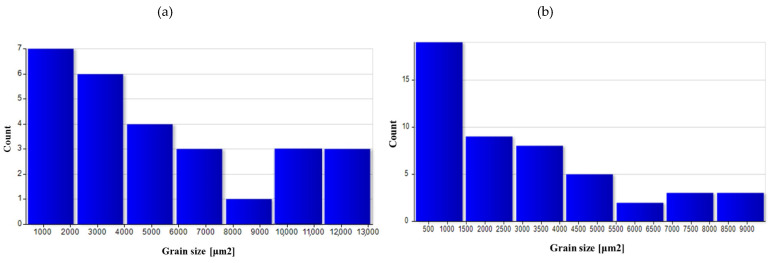
Grain surface distributions for the analyzed areas marked in Figure 8: (**a**) preform I as-cast in area 1, (**b**) preform I after homogenization in area 1.

**Figure 27:**
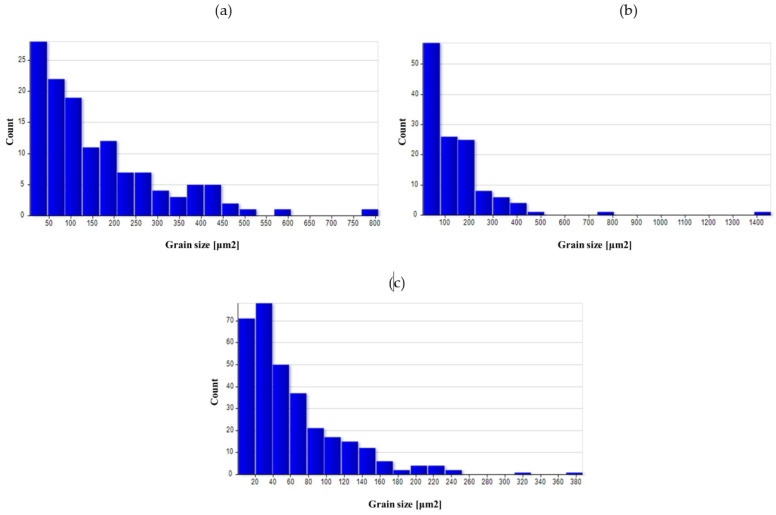
Grain surface distributions for the analyzed areas marked in Figure 8: (**a**) forging I area 1, (**b**) forging I area 2 (**c**) forging I area 3.

**Figure 28:**
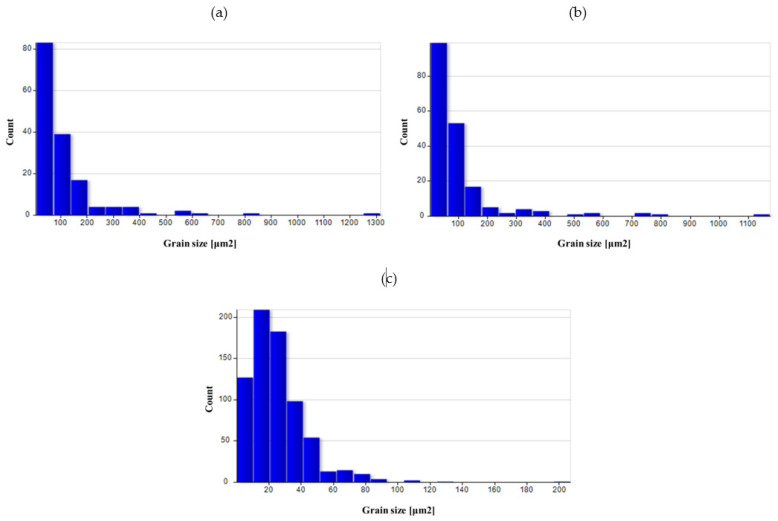
Grain surface distributions for the analyzed areas marked in Figure 8: (**a**) forging II area 1 (**b**) forging II area 2 (**c**) forging II area 3.

**Figure 29:**
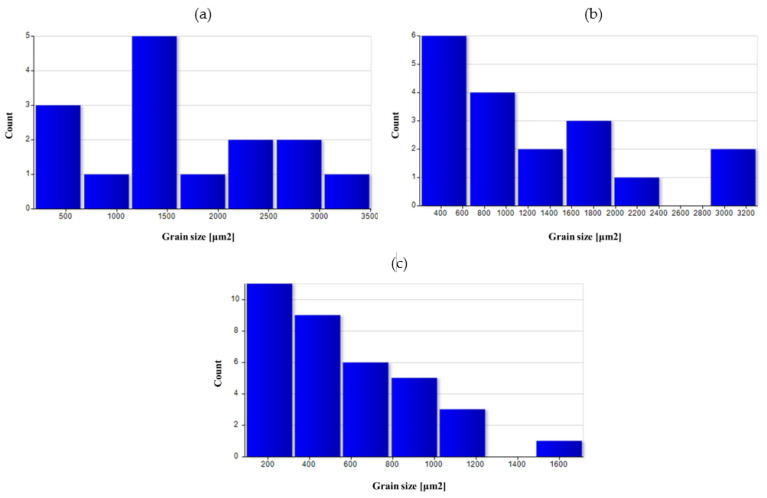
Grain surface distributions for the analyzed areas marked in Figure 8: (**a**) forging I heat-treated area 1, (**b**) forging I heat-treated area 2, (**c**) forging I heat-treated area 3.

**Figure 30:**
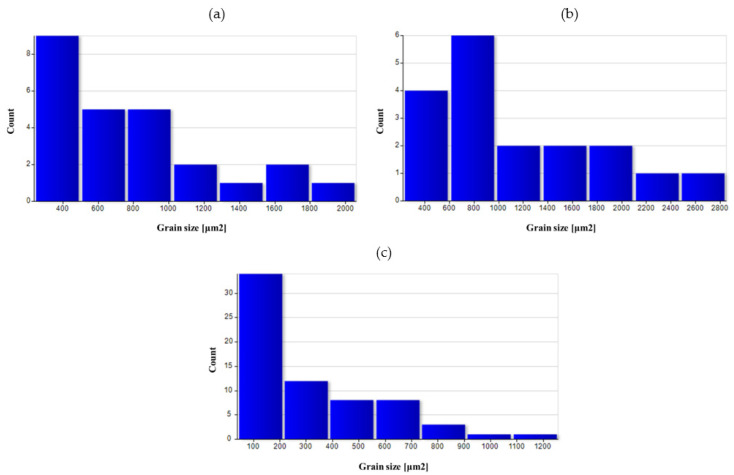
Grain surface distributions for the analyzed areas marked in Figure 8: (**a**) forging II heat-treated area 1, (**b**) forging II heat-treated area 2, (**c**) forging II heat-treated area 3.

Figure 26, Figure 27, Figure 28, Figure 29 and Figure 30 show grain surface distributions for the analyzed areas marked in Figure 8.

The analysis of the grain size distribution in individual areas showed that in most cases, the smallest grains were the most numerous group and that there were single grains of a much larger size. In the case of forged samples, for variant I in areas 1 and 2, most of the grains had a surface area below 100 μm^2^, and for area 3, below 60 μm^2^. For variant II in areas 1 and 2, the values were analogous, and in area 3, most of the grains were smaller than 40 μm^2^. After the heat treatment, the grain size increased and the differences in grain size in the individual areas reduced. Table 6 presents the results of measurements of the specific conductivity of the examined metallographic polished sections. The obtained resulted prove that after carrying out the heat treatment process in the form of homogenization on castings, the values of electrical conductivity decrease. Consequently, the increase in conductivity after the forging process from preforms could be detected. The highest conductivity for the considered variants of the process was shown by forgings after the forging and heat treatment, which verified that the heat treatment was carried out correctly.

**Figure 31:**
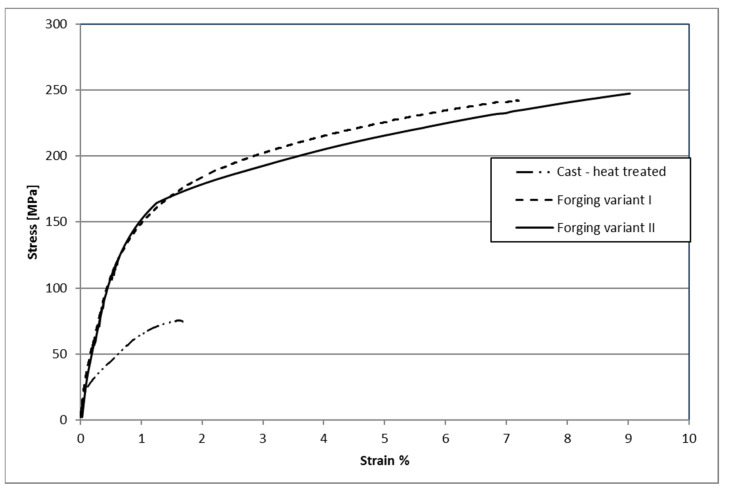
Example curves obtained during the static tensile test of samples made of AZ61 alloys.

Figure 31 shows example curves obtained during the static tensile test on the testing machine of samples made of AZ61 alloys. The analysis of the curves obtained in the static tensile test showed that the samples made of castings and subjected to the heat treatment process showed the smallest plasticity and tensile strength. The forgings after heat treatment were characterized by much higher strength and elongation. There were no significant differences between the samples forged using the lower and the higher preforms. The minimally higher values were in forging forged from a preform with a higher degree of forging.

Table 7 shows the results of measuring the hardness of samples made of AZ61 magnesium alloy for each of the tested variants. The hardness measurements show that in the case of AZ61 alloy, there was a slight but noticeable decrease in hardness after homogenization. As a result of forging, the hardness was increased to slightly lower than that of the casting. After thermal treatment, the hardness of the alloy increased significantly. No noteworthy differences were observed between the hardness of castings with different geometries (preform I and preform II) when using different billet. Finally, the hardness of the hammer-forged forgings of the second variant turned out to be the highest.

## 4. Summary and Conclusions

Based on the analyses conducted, the following conclusions were formulated:

- The objective of this study was to investigate the formability of AZ61 cast magnesium alloy using the example of an innovative technique for producing forging of aircraft mounts by hammer forging from cast preforms. The numerical and experimental results demonstrate that AZ61 cast magnesium alloy in sand molds has good formability in the hammer die forging process, and the proposed new forging method is a viable way of producing forgings of aircraft mounts with the required shape. The new method ensures considerable material and energy savings and higher properties of the product than previously applied techniques, consisting of forming aircraft mounts only from casts by machining operations.

The developed new technology of forging from preforms assumes greater production efficiency due to the shortening of the time of forging in one forging operation compared to the multi-stage forging from the extruded rod used in the industry.

- Structural studies have shown that the material’s structure undergoes substantial evolution at different stages of the process. Homogenization results in obtaining a consistent initial structure for the die forging process. After forging, significant grain refinement was observed, which is not uniform throughout the forging volume, but the grain size differences are perfectly acceptable. Further heat treatment makes the grain sizes and shapes uniform and allows the alloy to be strengthened by secondary precipitation. The conducted tests and conductivity analysis confirm the observed evolution of the material’s microstructure during the process. The detected decrease in hardness promotes forming, while its significant increase after heat treatment has a positive effect on the performance of forgings. Small differences in the values obtained for variants I and II may result, first of all, from the different cooling kinetics of castings with different volumes and the different degree of forging. Finally, variant II has higher mechanical properties and conductivity, but the differences are small. Comparing the maximum values of the strength and hardness properties of the new forging technology from forgings to the traditional rod forging technology, they are as follows: new technology-Rm = 250 MPa, HV = 75.5; bar forging-Rm = 292 MPa, HV = 65.7 [35].
- Lightweight magnesium alloys belong to the group of construction materials considered key importance for the future. Therefore, it is necessary to conduct further research developing the technique for manufacturing magnesium alloy products using forging hammers that have significantly higher output capacity than hydraulic presses. The positive results of previous studies on the hammer forging process from AZ61 magnesium alloy-shaped cast preforms prove that the research should be continued for other workable magnesium alloys.

## Figures and Tables

**Table 1:** Chemical composition of AZ61 magnesium alloy used in the experiment (wt %).

Al	Zn	Mn	Fe	Si	Cu	Ni	Mg
5.8–7.2	0.4–1.5	0.15–0.5	max 0.005	max 0.10	max 0.05	max 0.005	rest

**Table 2:** Conditions in the individual baths used for the etching process.

Bath	Conditions
I	Warm water, 50–70 °C
II	Cold water
III	Nitric acid aqueous solution HNO_3_ (around 20–35%)
IV	Cold water
V	Sodium hydroxide aqueous solution NaOH (around 10%) 50–70 °C

**Table 3:** Comparison of the volume and material losses of forging from cast preforms with the technology of forging from an extruded rod.

Volumes and Material Loss Analyzed	Forging	Billet in the Form of Extruded Rod	Billet in the Form of Cast Preform Variant I	Billet in Form of Cast Preform Variant II
Volume (mm^3^)	156,638.4	254,469	208,720.74	237,770.5
Volume of flash (mm^3^)		97,830.6	52,082.34	81,132.1
Material loss (%)		62.5	33.3	51.8
Decrease in material loss (%)			47	18

**Table 4:** Results of microanalysis of chemical composition by EDS method.

Scheme 1	Area	Element	Measurement Number			Mean (Weight Conc.)
			1	2	3	
Preform variant I as-cast	αMg matrix	Mg	89.45	89.33	-	89.39
		Al	7.38	7.09		7.24
		Zn	3.17	3.58		3.38
	β-Intermetallic	Mg	52.34	52.32	-	52.33
		Al	31.22	30.94		31.08
		Zn	16.44	16.74		16.59
	Eutectic	Mg	49.95	54.84	-	52.40
		Al	30.91	28.44		29.68
		Zn	19.14	16.72		17.93
Preform variant I as-castafter homogenization	Homogenized matrix	Mg	91.42	92.07	91.96	91.75
		Al	7.08	6.39	6.44	6.64
		Zn	1.50	1.55	1.61	1.55

**Table 5:** Average grain size in the studied areas marked in Figure 8.

Sample	Mean Grain Size [μm^2^] in Area:		
	1	2	3
Preform I as-cast	5300.6	-	-
Preform I after homogenization	2849.6		
Forging I	159.1	140.7	61.2
Forging II	108.8	101.1	25.3
Forging I heat-treated	1676.9	1216.6	561.3
Forging II heat-treated	793.3	1169.7	315.9

**Table 6:** Results of specific conductivity s measurements and average grain diameter.

Sample	Measurement Number			Mean (MS/m)	Standard Deviation
	1	2	3		
Preform I after casting	8.17	8.12	8.4	8.22	0.15
Preform I after homogenization	7.46	7.39	7.46	7.43	0.04
Forging I after forging	8.39	8.52	8.39	8.43	0.07
Forging I after forging and heat treatment	9.05	8.58	9.01	8.88	0.26
Preform II after casting	9.01	9.92	8.97	8.99	0.09
Preform II after homogenization	7.86	7.76	7.80	7.80	0.05
Forging II after forging	8.28	8.62	8.00	8.30	0.31
Forging II after forging and heat treatment	9.05	9.10	8.97	9.04	0.06

**Table 7:** Results of hardness measurement for samples made of AZ61 alloy.

Sample	Measurement Number			Mean (HV05)	Standard Deviation
	1	2	3		
Preform I after casting	65.1	71.2	67.5	69.9	2.5
Preform I after homogenization	56.2	54.7	55.5	55.5	0.6
Forging I after forging	62.6	62.2	64.2	63.0	0.9
Forging I after forging and heat treatment	73.6	74.4	72.1	73.4	1.0
Preform II after casting	68.6	69.1	67.3	68.3	0.08
Preform II after homogenization	52.2	56.7	57.4	55.4	2.3
Forging II after forging	62.1	65.5	63.1	63.6	1.4
Forging II after forging and heat treatment	77.7	73.9	74.9	75.5	1.6

## Data Availability

The data presented in this study are available on request from the corresponding author. The data are not publicly available due to protection of intellectual property.
